# The Hermans–Rasson test as a powerful alternative to the Rayleigh test for circular statistics in biology

**DOI:** 10.1186/s12898-019-0246-8

**Published:** 2019-08-07

**Authors:** Lukas Landler, Graeme D. Ruxton, E. Pascal Malkemper

**Affiliations:** 1 Research Institute of Molecular Pathology (IMP), Vienna Biocenter (VBC), Campus-Vienna-Biocenter 1, 1030 Vienna, Austria; 2 School of Biology, University of St Andrews, St Andrews, KY16 9TH UK

**Keywords:** R functions, Animal navigation, Migration, Emlen funnel, Behaviour, Biostatistics, Circadian, Chronobiology

## Abstract

**Background:**

Circular data are gathered in diverse fields of science where measured traits are cyclical in nature: such as compass directions or times of day. The most common statistical question asked of a sample of circular data is whether the data seems to be drawn from a uniform distribution or one that is concentrated around one or more preferred directions. The overwhelmingly most-popular test of the null hypothesis of uniformity is the Rayleigh test, even though this test is known to have very low power in some circumstances. Here we present simulation studies evaluating the performance of tests developed as alternatives to the Rayleigh test.

**Results:**

The results of our simulations demonstrate that a single test, the Hermans and Rasson test is almost as powerful as the Rayleigh test in unimodal situations (when the Rayleigh test does well) but substantially outperforms the Rayleigh test in multimodal situations.

**Conclusion:**

We recommend researchers switch to routine use of the new Hermans and Rasson test. We also demonstrate that all available tests have low power to detect departures from uniformity involving more than two concentrated regions: we recommend that where researchers suspect such complex departures that they collect substantially-sized samples and apply another recent test due to Pycke that was designed specifically for such complex cases. We provide clear textual descriptions of how to implement each of these recommended tests and encode them in R functions that we provide.

**Electronic supplementary material:**

The online version of this article (10.1186/s12898-019-0246-8) contains supplementary material, which is available to authorized users.

## Background

In many branches of science, data is collected on scales that are cyclical. The two most obvious cases of this relate to times and directions. For example, we might collect data on the time of day that calls are made to the emergency services, or the occurrence of homicides in relation to phases of the moon, or particle counts of water samples from the surface waters of a lake at different times of year. In all these cases there is a cyclical nature to the data—we might label December the 12th month, but it is intrinsically closer to the 1st month of the next year than the 9th month of the current year. Directions can have a similar cyclical nature, if we measure for example, the directions relative to the shortest path to their loft that homing pigeons take after release, or the bond angles of molecules during collisions with the walls of a container, or the directions relative to true North that resting fish adopt. Such data requires different treatment than data collected on a linear scale (e.g. lengths or masses). A number of texts provide an introduction to the analysis of such circular data (so called because you could readily envisage the data points on a scale encapsulated as the circumference of a circle): e.g. [1, 3, 7, 9, 12, 14].

The most common question that is asked of a sample of such data is whether the data is aggregated into one or more “preferred” directions. Within the framework of null hypothesis statistical testing, this equates to testing the null hypothesis that the underlying distribution from which the sample is drawn is uniformly spread around the full circumference of the circle, so that no direction is inherently preferred over any other. Although model fitting approaches are entirely appropriate (see [4] for an excellent overview), null hypothesis statistical testing remains the norm amongst those investigating circular data, and the null hypothesis of uniformity is almost always tested in any statistical examination of a sample of circular data. Further, it is almost always tested using what is called the Rayleigh test (originally due to [16] but also defined and discussed in all the general texts listed above). There can be good reasons for adopting this test. It can be shown (e.g. [18]) to be the optimal test if the data is continuously distributed and any departure from uniformity takes a von Mises form (a symmetrical, unimodal distribution often referred to as the circular analogue of a normal distribution). Further, this test also can be demonstrated numerically to perform very reliably when deviations are of other unimodal forms [8] or if data is discrete (e.g. as might be produced by a measuring instrument with finite precision; [6]). However, this test is known to be less reliable when the deviation from uniformity is multi-modal, specifically its power to reject the null hypothesis when the deviation from uniformity involves more than one mode can be concerningly low even for substantial sample sizes [1, 17]. We recently demonstrated this for a broad range of multimodal distributions [8]. This should not be seen as a failing or weakness of the Rayleigh test, since it was not originally designed to detect deviations other than unimodal von Mises ones. In [8], we highlighted a test due to Hermans and Rasson [5] that showed considerably more power than the Rayleigh test to detect some types of multimodal deviations, however we did not explore its performance for unimodal distributions. We argued that the power gain in multimodal scenarios should encourage more widespread uptake of the Hermans–Rasson test. Indeed, unless the researcher is certain that only von Mises deviations from uniformity are possible or of interest, they might be best served by using this test in preference to the Rayleigh test. The test we use in our recent analysis was an improved version (referred to as HR in this paper) in comparison to the original proposal (HR∞), which Hermans and Rasson [5] themselves and Pycke [15] implied as having generally greater power. Here we explore the robustness of this advice by comparing the Rayleigh, HR∞ and HR tests in further sets of simulations. We also explore whether the HR test is really the optimum one to recommend for widespread replacement of the Rayleigh test, since this has been subject to relatively little previous examination [5, 8, 15]. Accordingly, we compare the performance of these tests with one other: Pycke [15] argued that the HR test produced good performance for one or two modes but for larger number of modes he offered his own test as being more powerful. We call this test the Pycke test.

## Results

**Fig. 1:**
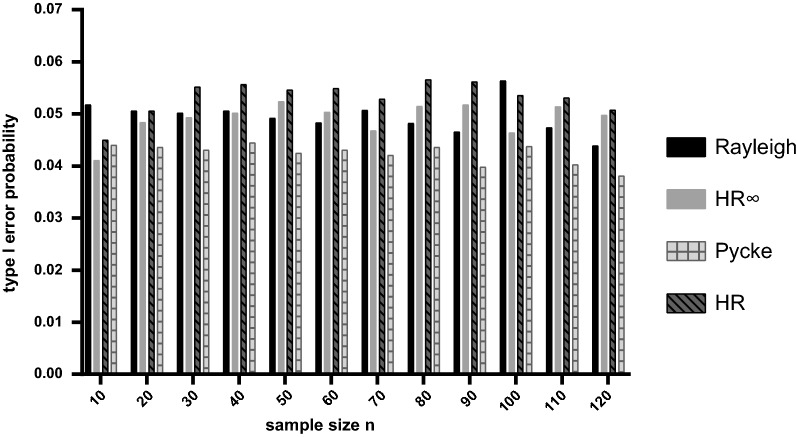
Estimated type I error rates for random samples of specified size drawn from a uniform population based on 10,000 replicates in each case. The significance level was set to 5%

When the underlying distribution from which samples were drawn was a uniform distribution then we found that all four tests maintained the Type I error rate at close to the nominal 5% value for a broad range of sample sizes (Fig. 1). The Type I error rate also stays close to the expected values when using 1% or 10% significance levels (Additional file 1: Figure S1).

**Fig. 2:**
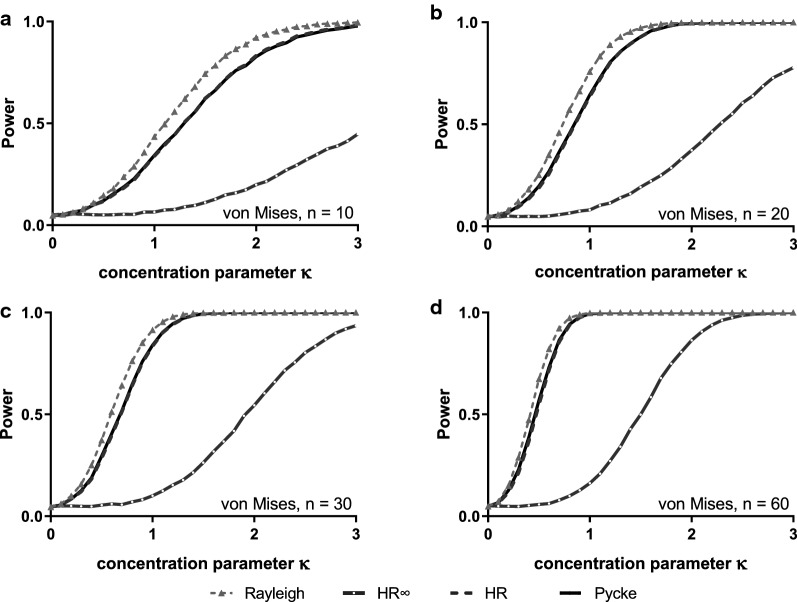
Estimated power for random samples of size **a** 10, **b** 20, **c** 30 or **d** 60 drawn from a von Mises distribution with a range of concentration parameters (κ)

Next, we considered the von Mises distribution, a symmetric unimodal distribution with a bell shape that often is described as the circular equivalent of the normal distribution (for examples of the different density distributions see [8], Fig. 2). The Rayleigh test is known to be theoretically optimal in this case, and our simulations agree with this in demonstrating that it has the highest overall power (Fig. 2). The HR and Pycke functions, however, have only slightly less power, with the difference only approaching non-trivial levels for the smallest sample sizes considered (n = 10). HR∞ is noticeably inferior to the other tests (in agreement with previous work). We found essentially similar results for an asymmetric unimodal distribution where the performance difference between the Rayleigh, HR and Pycke tests was even smaller (the wrapped skew normal distribution: see for example [14] for details, Additional file 1: Figure S2). Note that while increasing values of *κ* for the von Mises distribution signify increasing concentration around the central value, for the wrapped skew normal distribution increasing the parameter ω corresponds to increased dispersion (i.e. decreasing concentration).

**Fig. 3:**
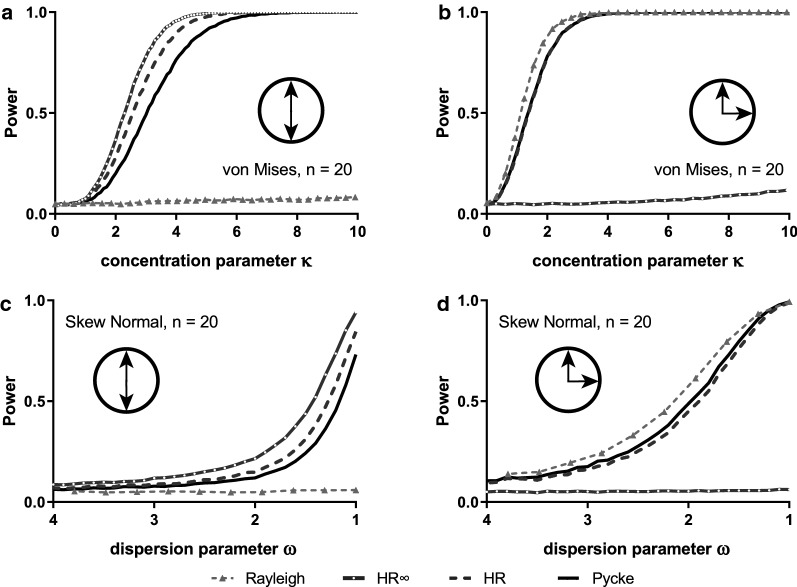
Estimated power of random samples (n = 20) drawn from bimodal distributions von Mises distributions [symmetrical: (**a**) and asymmetrical: (**b**)] and from bimodal wrapped skew normal distributions [symmetrical: (**c**) and asymmetrical: (**d**)], with a range of concentration/dispersion parameters. That is, for **a** the sample is drawn from an underlying distribution made of up two identical von mises distributions with central values positioned a half circle away from each other; **c** is the same but using wrapped skew normal distributions. **b** Is like **a**, and **d** is like **c** except that the two distributions are now only a quarter circle apart

In a multimodal situation with a combination of two identical von Mises distributions placed symmetrically opposite each other on the circle, the Rayleigh test performed poorly, while all the other tests performed well (Fig. 3a). When the two distributions were a quarter circle apart, all the tests perform well except for HR∞ (Fig. 3b). Analyses of similar situations using wrapped skew normal distributions revealed very similar behaviour of the different tests (Fig. 3c, d). In all multimodal situations, if not stated otherwise, the densities were equally distributed between the modes (e.g. 0.5 for each mode in a bimodal situation), meaning that concentration changes affect each mode equally. When the density of the two distributions was set to be unequal (0.75, 0.25), the overall power of the tests, in particular of the Rayleigh test, increased, but the general trends remained the same (Additional file 1: Figure S3).

**Fig. 4:**
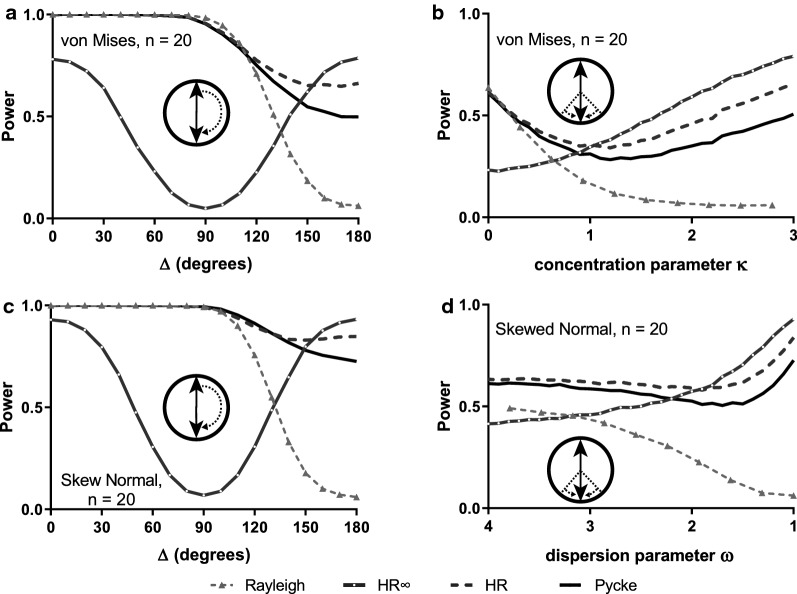
Estimated power of random samples (n = 20) drawn from bimodal distributions. In **a**, **c** we vary the central concentration points of the two identical constituent distributions (changing from exactly coincident with each other at the left extreme to exactly opposite each other at the right). In **a** the two distributions are von Mises with *κ* = 3; in **c** they are wrapped skew normal distributions with ω = 1. In **b**, **d** the two constituent distributions are at opposite points on the circle but now their concentration parameters differ, one is fixed, the value of the other distribution given on the x-axis. In **b** the two distributions are von Mises with *κ* = 3 for the fixed value; in **d** they are wrapped skew normal distributions with ω = 1 for the fixed value

When we varied the angle between the central concentrations of the two constituent identical unimodal distributions, from completely coincident (i.e. collapsing to the unimodal case) to completely opposite (yielding a symmetric bimodal aggregated distribution), we found low performance with HR∞ when coinciding and low performance of the Rayleigh test in the completely opposite case. The other two tests show good performance across the whole range (with HR being superior to Pycke) (Fig. 4a, c). It is not surprising that performance is lower in general towards the right side of these figure panels. For such small sample sizes especially, detecting unimodal departures from uniformity is simply less challenging than detecting multimodal departures where there is necessarily a greater spread of sampled values around the circle. We observed similar relative performance when we varied the concentration of the two constituents in making up the aggregated distribution (Fig. 4b, d). Again, the results of von Mises distributions (Fig. 4a, b) and wrapped skew normal distributions (Fig. 4c, d) follow the same general trend. The same trends with overall higher power are visible at a larger sample size (n = 60) (Additional file 1: Figure S4).

**Fig. 5:**
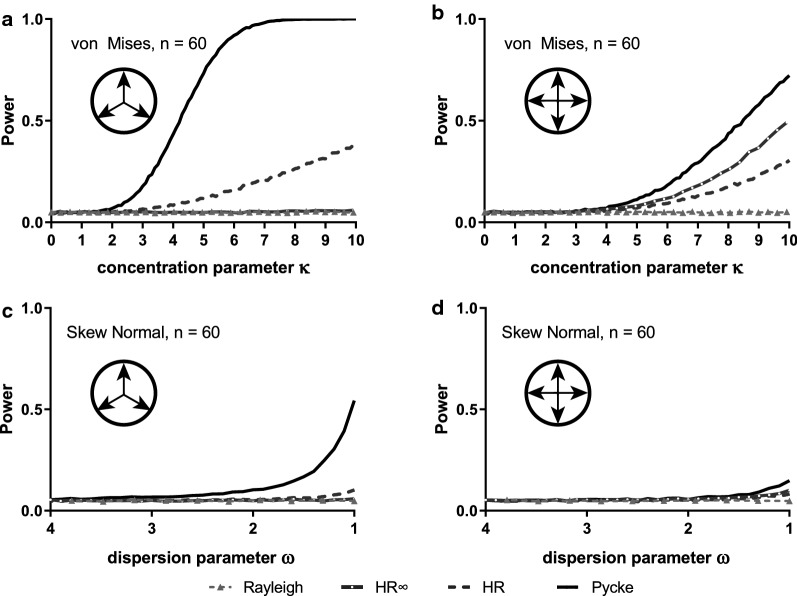
Estimated power of random samples (n = 60) drawn from multimodal Von Mises distributions [3 symmetrical modes: (**a**) and 4 symmetrical modes: (**b**)] and from multimodal wrapped skew normal distribution [3 symmetrical modes: (**d**) and 4 symmetrical modes: (**d**)]

We further explored symmetrical multimodal situations; for either three or four modes and either von Mises or wrapped skew normal constituent distributions (Fig. 5). Essentially, our results demonstrate that detecting departures from uniformity is very challenging in this case even at large sample size (n = 60), with only the Pycke test showing useful levels of power and then only for the trimodal case when the constituent distributions are very concentrated (Fig. 5a). At a small sample size (n = 20) power is exceptionally low, and again only the Pycke test offers useful power and only when the sample is tightly concentrated around the modes (Additional file 1: Figure S5). Changing the density of one of the distributions while keeping the others identical (creating unequal distributions), increased the overall power, but the general trends remained the same (Additional file 1: Figure S6).

## Discussion

We have previously presented comparison by simulation of a more extensive battery of alternative tests for testing the null hypothesis of uniformity of circular data [8]. Our simulations here allow us to significantly improve on the conclusions provided in that work, which were mainly based on the analysis of tests that are available in statistical software packages (Watson’s test, Kuiper’s test and Rao’s spacing test). The Rayleigh test was found to be superior to them, hence their omission from the current work. In Landler et al. [8] we recommended continuing use of the Rayleigh test when unimodal departure from uniformity is expected, and the HR test for multimodal departures. We evaluated another modern test due to Bogdan et al. [2] as an alternative to the HR test but found that its performance was generally inferior, hence we did not consider it further in this study. However, importantly, in our previous work we had not evaluated the power of the HR in situations with expected unimodal departures. Here, we performed that evaluation and demonstrate that the performance of this test in detecting unimodal departures is only slightly inferior to the Rayleigh test. Further, we had previously not considered the test due to Pycke [15] which was specifically designed for cases with more than two modes. We found it to perform better than the other tests in such cases, but only in situations with highly concentrated data.

In summary our analyses show that for researchers interested in testing for departure from uniformity, the HR function represents an unjustly neglected tool. The simulations presented here and in Landler et al. [8] highlight that this test offers as good control of type I error as currently-popular methods, combined with power to detect a wide range of unimodal and bimodal distributions that appears to be overall superior to any alternative method including the Rayleigh test. Although the Rayleigh test can offer slightly superior power for univariate departures, the difference in power is never substantial, whereas the HR test outcompetes other tests (including the Rayleigh test) for bimodal departures and can often offer markedly superior power. We could construct no unimodal or bimodal distribution for which this test was substantially outcompeted by any other. Thus, researchers can gain a power advantage by selecting this test routinely when they are interested in departures from normality that might be unimodal or bimodal.

Although the Rayleigh test is currently by far the most popular test, we and others (e.g. [1, 8, 15, 17]) have demonstrated that there are situations where its power is exceptionally low. We can find no analogous unimodal or bimodal situations for the HR test—so we believe that routine replacement of the Rayleigh test with the HR test would benefit the field of statistical analysis of circular data.

Our simulations highlight that rejection of non-uniformity is very challenging in situations where the possible number of modes is greater than two and their distribution is unknown. In such situations we recommend that researchers strive to maximise the size of the sample they obtain and apply the Pycke test only if their underlying knowledge of the system gives them reason to think that sample points will be tightly concentrated around the modes. In other circumstances, no test offers sufficiently useful power to be recommended.

## Conclusions

We present simulations that demonstrate the superior power of the HR and Pycke tests in particular in multimodal situations. We provide the R functions for the two recommended tests, which now can be easily implemented. Considering the increasing popularity of R in the research community we believe this tool is highly useful for all researchers working with circular data. Furthermore, we hope that our analysis also provides compelling cause for other software developers to include these tests in their new software versions and library functions. This could lead to the wider uptake of such very useful statistical procedures.

## Methods

### Defining the four different tests that we compare

The Rayleigh test is defined and discussed in all the general texts listed above (we recommend [3] for a particularly clear discussion), and readily available in a number of circular statistics packages. Here we use the implementation in the function *Rayleigh.test* in the *R* package *circular*.

For the three remaining tests it is difficult to give a concise heuristic motivation for the form of the test statistics, although they are grounded in the theory of decomposing the description of any shape using Fourier series. The interested and mathematically-confident reader should consult Hermans and Rasson [5], Bogdan et al. [2], Pycke [15] for full discussions. Further, none of these tests are currently available in any software package. However, their calculations are numerically intensive but relatively simple to describe. Here we provide mathematical definitions of each test, and in the additional information we encode each of these definitions within R functions for data in radians as well as in degrees (see Additional file 2).

In each case, the p-value of the test must be obtained by simulation. We use the following methodology. First the value of a given test statistic is calculated for the sample of interest. We then draw a number *m* (9999) of pseudo-samples each of size *n* (the size of the original sample) from a uniform distribution on [0,2π). We then calculate the value of the test statistic for each of these pseudo-samples. We next calculate the number of pseudo-samples that give a test statistic equal to or greater in magnitude to that of the original sample, call that number Q. Then the p-value of the test is given by (Q + 1)/(*m* + 1). See [11] for a full discussion of the theoretical underpinning of this methodology. To fully define each test now, we need only describe how to calculate the test statistic for any sample.

In each case we assume that we have a sample of size *n* containing values {α_1_,…,α_n_} in radian measure in the range [0,2π).

For HR∞ test, the test statistic *T* is described in particularly clear form by Bodgan et al. [2]:

$$T = \left( {\frac{n}{\pi }} \right) - \left( {\frac{1}{2n}} \right)\mathop \sum \limits_{i = 1}^{n} \mathop \sum \limits_{j = 1}^{n} \left| {sin\left( {\alpha_{i} - \alpha_{j} } \right)} \right|$$

For HR, the clearest description of the test statistic *V* is given by [15]:

$$V = \left( {\frac{1}{n}} \right)\mathop \sum \limits_{i = 1}^{n} \mathop \sum \limits_{j = 1}^{n} \left( {\left| {\left| {\alpha_{i} - a_{j} } \right| - \pi } \right| - \frac{\pi }{2} - 2.895\left( {\left| {sin\left( {\alpha_{i} - \alpha_{j} } \right)} \right| - \frac{2}{\pi }} \right)} \right)$$

For the Pycke test the test statistic *V* is given by

$$V = \left( {\frac{1}{n}} \right)\mathop \sum \limits_{i = 1}^{n} \mathop \sum \limits_{j = 1}^{n} \left( {\frac{{2\left( {cos\left( {\alpha_{i} - a_{j} } \right) - \sqrt {0.5} } \right)}}{{1.5 - \left( {2\sqrt {0.5} cos\left( {\alpha_{i} - \alpha_{j} } \right)} \right)}}} \right)$$

## General methods

We evaluate the relative performance of the different tests by simulation in *R*, reporting the fraction of 10,000 samples of fixed size drawn from particular parent populations for which the test reported a p-value less than 0.05. For sample generation we used the rcircmix() function from the NPCirc package in R (see Additional file 2 “Section 3” for code to generate the parent populations) [13].

We used our own code (see Additional file 2) for all the tests except the Rayleigh test for which we used the function *rayleigh.test* in package *circular* in *R* [10]. We define particular parent populations in the relevant sections of “Results” section and relevant figure legends. If not stated otherwise the proportion in multimodal distributions was equal between all modes. In case of unequal proportions, we used 0.25 and 0.75 for bimodal, 0.6, 0.2 and 0.2 for trimodal, and 0.4, 0.2, 0.2 and 0.2 for the quadramodal case.

In order to calculate the power of a given test we drew 10,000 random samples from the distribution of interest [either Von Mises or wrapped skew normal (skewness = 30)] and applied each of the tests. We then calculated statistical power (i.e. proportion of tests with p < 0.05). This general approach was used for all the simulations.

## Additional files

**Additional file 1.** Additional figures S1–S6.

**Additional file 2.** R code.

## Data Availability

Most R code used to generate the datasets is provided in the additional information of this article. R scripts not presented in the current study are available from the corresponding author on reasonable request.

## Abbreviations

HR: Hermans–Rasson test (new version)
HR∞: Hermans–Rasson test (original version)
