# Supplementary material for: The Hermans–Rasson test as a powerful alternative to the Rayleigh test for circular statistics in biology
Source: BMC Ecol. 2019 Aug 7;19:30. doi: 10.1186/s12898-019-0246-8 (PMC6686250; doi:10.1186/s12898-019-0246-8)
Supplement: Supplementary file 1 — Additional file 1. Additional figures S1–S6. [file 12898_2019_246_MOESM1_ESM.pdf]

Additional file 1: Additional figures S1-S6

BMC Ecology

The Hermans-Rasson test as a powerful alternative to the Rayleigh test for circular statistics in biology

Lukas Landler[1], Graeme D. Ruxton[2], E. Pascal Malkemper[1]

Affiliations

[1]Research Institute of Molecular Pathology (IMP), Vienna Biocenter (VBC), Campus-Vienna-Biocenter 1, 1030 Vienna, Austria

[2]School of Biology, University of St Andrews, St Andrews KY16 9TH, UK

Corresponding author email address: pascal.malkemper@imp.ac.at

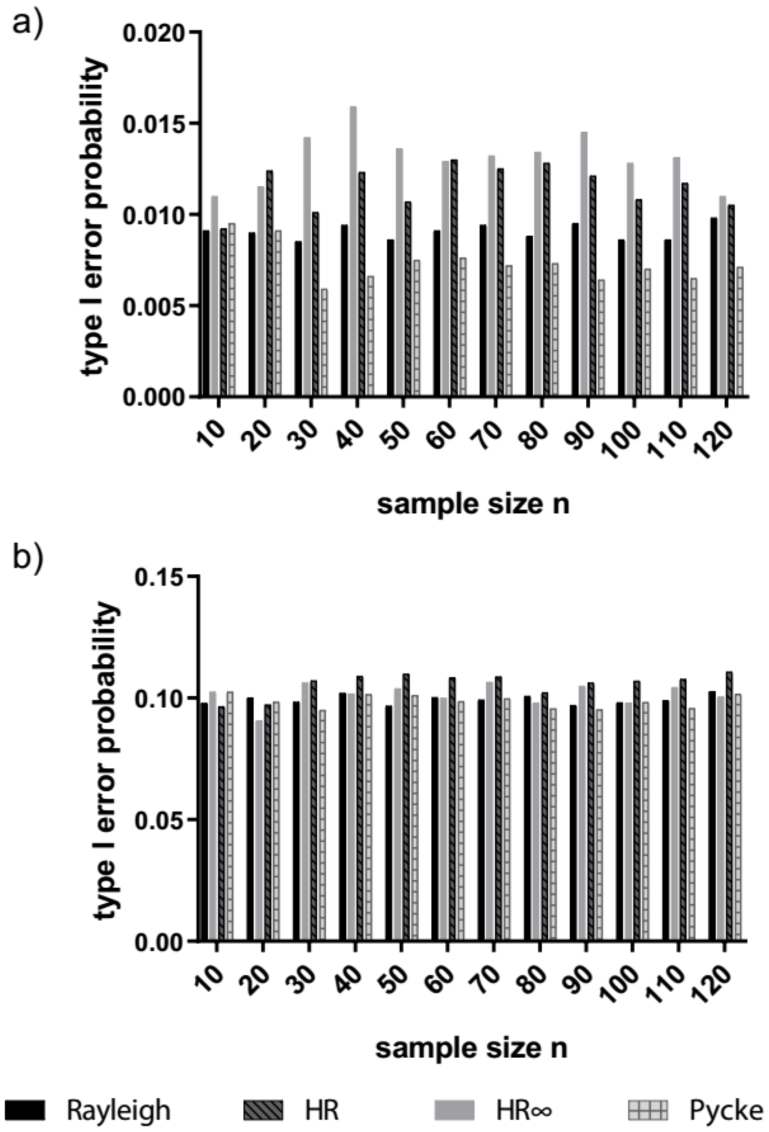

Figure S1: Estimated type I error rates for random samples of specified size drawn from a uniform population based on 10,000 replicates in each case. The significance level was set to 1% in a) and 10% in b).

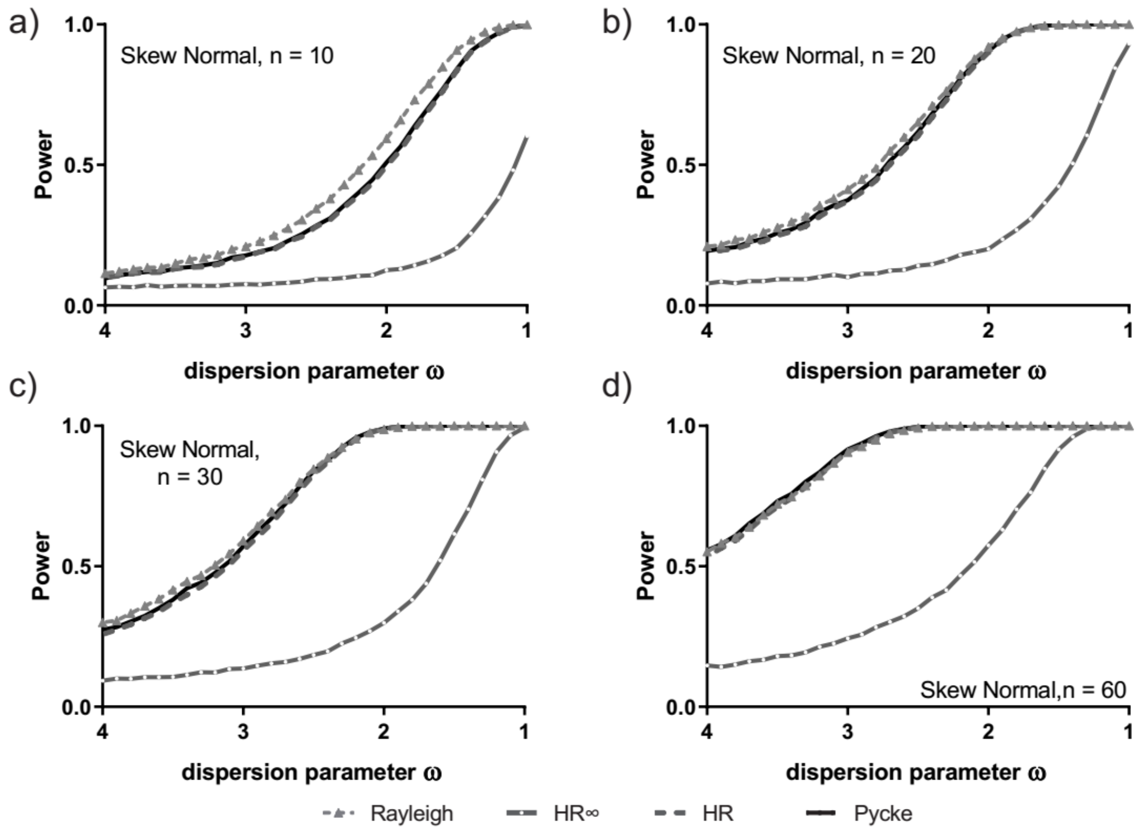

Figure S2: Estimated power of four random samples of size (a) 10, (b) 20, (c) 30 or (d) 60 drawn from wrapped skew normal distributions (with skewness parameter = 30 throughout) with a range of dispersion parameters (ω).

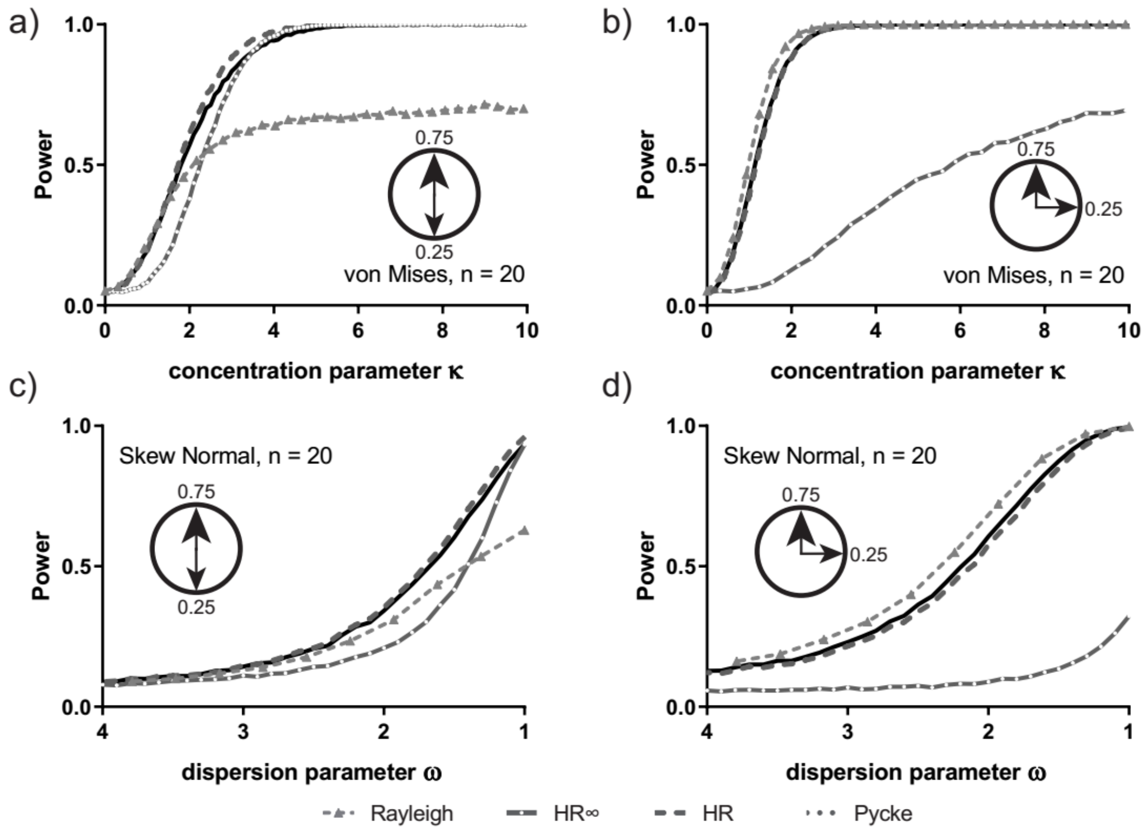

Figure S3: Estimated power of random samples (n = 20) drawn from bimodal von Mises distributions with unequal densities between the two modes (symmetrical: (a) & asymmetrical: (b)) and from bimodal wrapped skew normal distributions (symmetrical: (c) & asymmetrical: (d)), with a range of concentration/dispersion parameters. That is, for (a) the sample is drawn from an underlying distribution made of up two unequal von Mises distributions with central values positioned a half circle away from each other; (c) is the same but using wrapped skew normal distributions. Panel (b) is like (a) and (d) is like (c) except that the two distributions are now only a quarter circle apart.

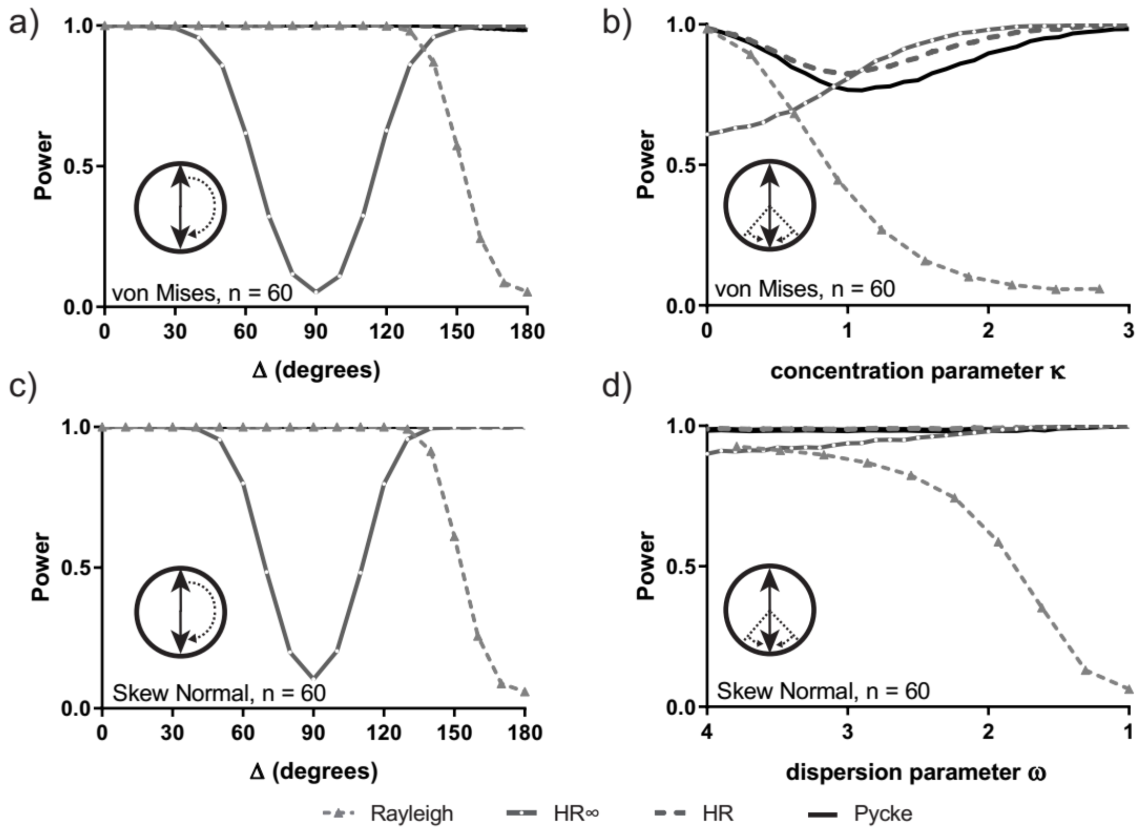

Figure S4: Estimated power of random samples (n = 60) drawn from bimodal distributions. In (a) & (c) we vary the central concentration points of the two identical constituent distributions (changing from exactly coincident with each other at the left extreme to exactly opposite each other at the right). In (a) the two distributions are von Mises with $\kappa = 3$; in (c) they are wrapped skew normal distributions with $\omega = 1$. In (b) & (d) the two constituent distributions are at opposite points on the circle but now their concentration parameters differ, one is fixed, the value of the other distribution given on the x-axis. In (b) the two distributions are von Mises with $\kappa = 3$ for the fixed value; in (d) they are wrapped skew normal distributions with $\omega = 1$ for the fixed value.

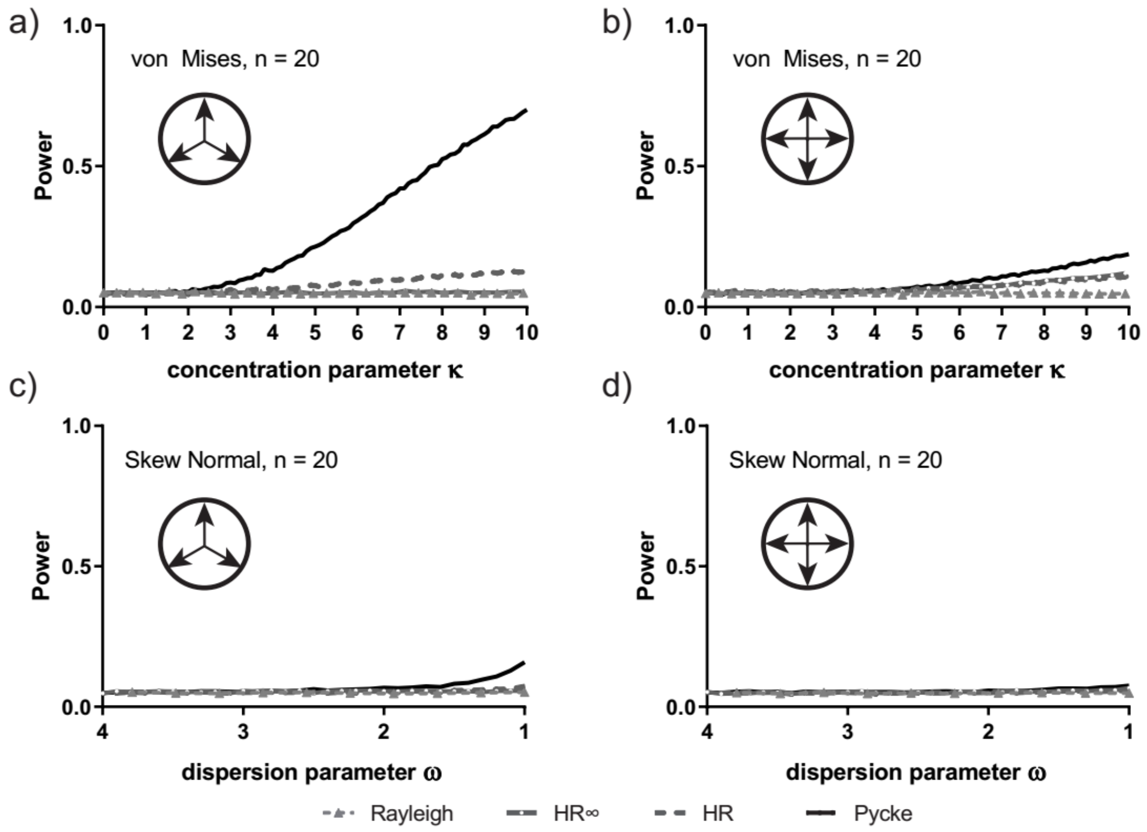

Figure S5: Estimated power of random samples (n = 20) drawn from multimodal von Mises distributions (3 symmetrical modes: (a) & 4 symmetrical modes: (b)) and from multimodal wrapped skew normal distributions (3 symmetrical modes: (d) & 4 symmetrical modes: (d)).

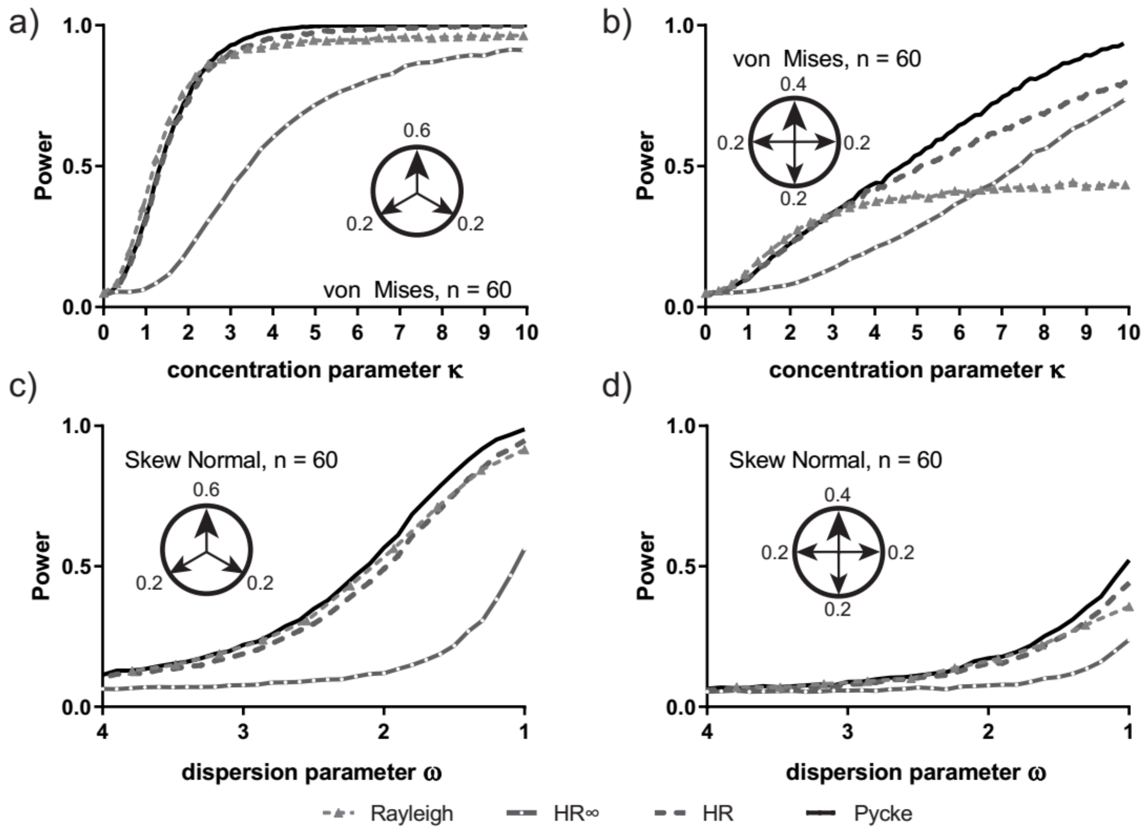

Figure S6: Estimated power of random samples (n = 60) drawn from multimodal von Mises distributions with unequal densities (3 symmetrical modes: (a) & 4 symmetrical modes: (b)) and from multimodal wrapped skew normal distributions (3 symmetrical modes: (d) & 4 symmetrical modes: (d)).
