# Supplementary material for: The Hermans–Rasson test as a powerful alternative to the Rayleigh test for circular statistics in biology
Source: BMC Ecol. 2019 Aug 7;19:30. doi: 10.1186/s12898-019-0246-8 (PMC6686250; doi:10.1186/s12898-019-0246-8)
Supplement: Supplementary file 2 — Additional file 2. R code. [file 12898_2019_246_MOESM2_ESM.pdf]

Additional file 2: R code

BMC Ecology

The Hermans-Rasson test as a powerful alternative to the Rayleigh test for circular statistics in biology

Lukas Landler[1], Graeme D. Ruxton[2], E. Pascal Malkemper[1]

Affiliations

[1]Research Institute of Molecular Pathology (IMP), Vienna Biocenter (VBC), Campus-Vienna-Biocenter 1, 1030 Vienna, Austria

[2]School of Biology, University of St Andrews, St Andrews KY16 9TH, UK

Corresponding author email address: pascal.malkemper@imp.ac.at

1. **R code for the self-written functions and an example of applying the tests to a circular data set - (input data in radians!!! – see page 4 for input in degrees)**

#loading library

library("circular")

#setting function for HR infinity (the originally proposed HR test referred to as HR∞ in the paper)

```
HermansRassonT <- function(sample){
        n <- length(sample)
        total <- 0
        for (i in 1:n){
            for (j in 1:n){total <- total + abs(sin(sample[i]-sample[j]))}}
        T <- abs((n/pi)-(total/(2*n)))
        return(T)}

HermansRassonP <- function(sample){
        univals <- 9999
        n <- length(sample)
        sample<-circular(sample, units ="radians")
        testset<- rep(0,univals)
        for (f in 1:univals){
            data1 <- matrix(rcircularuniform(n,
control.circular=list(units="radians")))
            testset[f] <- HermansRassonT(data1)}
        Tsample <- HermansRassonT(sample)
        counter <- 1
        for(j in 1:univals){if(testset[j]>Tsample){counter <- counter+1}}
        p <- counter/(univals+1)
        return(p)}
```

#setting function for HR (a new version of the HR test referred to as HR in the paper)

```
HermansRasson2T <- function(sample){
        n <- length(sample)
```

```
		total <- 0
		for (i in 1:n){
			for (j in 1:n){ total <- total + abs(abs(sample[i]-sample[j])-pi)-
(pi/2)
	total <- total - (2.895*(abs(sin(sample[i]-sample[j]))-(2/pi)))}}
		T <- total/n
		return(T)}
HermansRasson2P <- function(sample){
		univals <- 9999
		n <- length(sample)
		sample<-circular(sample, units ="radians")
		testset<- rep(0,univals)
		for (f in 1:univals){
			data1 <- matrix(rcircularuniform(n,
control.circular=list(units="radians")))
			testset[f] <- HermansRasson2T(data1)}
		Tsample <- HermansRasson2T(sample)
		counter <- 1
		for(j in 1:univals){if(testset[j]>=Tsample){counter <- counter+1}}
		p <- counter/(univals+1)
		return(p)}
```

#Setting function for Pycke test

```
PyckeT <- function(sample){
		n <- length(sample)
		total <- 0
		for (i in 2:n){
			for (j in 1:(i-1)){
				numerator <- cos(sample[i]-sample[j])-sqrt(0.5)
				denominator <- 1.5-(2*sqrt(0.5)*cos(sample[i]-
sample[j]))
				total <- total + (numerator/denominator)}}
		T <- (2*total)/(n-1)
		return(T)}

PyckeP <- function(sample){
		univals <- 9999
```

```
        n <- length(sample)
        sample<-circular(sample, units ="radians")

        testset<- rep(0,univals)
        for (f in 1:univals){
                data1 <- matrix(rcircularuniform(n, control.circular=list(units="radians")))
                testset[f] <- PyckeT(data1)}
        Tsample <- PyckeT(sample)
        counter <- 1
        for(j in 1:univals){if(testset[j]>=Tsample){counter <- counter+1}}
        p <- counter/(univals+1)
        return(p)}
```

```
#Preparing a test sample (von Mises) with a given sample size "n" and kappa
n <- 12
kappa <- 0.8
sampletest <- rvonmises(n, circular(0), kappa, control.circular=list(units="radians"))

#Applying the rayleigh test
rayleigh.test(sampletest)

#applying the HR infinity test
HermansRassonP(sampletest)

#applying the HR test
HermansRasson2P(sampletest)

#applying the Pycke test
PyckeP(sampletest)
```

2. **R code for the self-written functions and an example of applying the tests to a circular data set – (input data in degrees!!!)**

#loading library

```
library("circular")
```

#setting function for HR infinity (the originally proposed HR test referred to as HR∞ in the paper)

```
HermansRassonT <- function(sample){
        n <- length(sample)
        total <- 0
        for (i in 1:n){
            for (j in 1:n){total <- total + abs(sin(sample[i]-sample[j]))}}
        T <- abs((n/pi)-(total/(2*n)))
        return(T)}

HermansRassonPDeg <- function(sample){
        univals <- 9999
        n <- length(sample)
        sample<-circular(sample)
        sample <- rad(sample)
        testset<- rep(0,univals)
        for (f in 1:univals){
            data1 <- matrix(rcircularuniform(n,
control.circular=list(units="radians")))
            testset[f] <- HermansRassonT(data1)}
        Tsample <- HermansRassonT(sample)
        counter <- 1
        for(j in 1:univals){if(testset[j]>Tsample){counter <- counter+1}}
        p <- counter/(univals+1)
        return(p)}
```

#setting function for HR (a new version of the HR test referred to as HR in the paper)

```
HermansRasson2T <- function(sample){
        n <- length(sample)
```

```
		total <- 0
		for (i in 1:n){
			for (j in 1:n){ total <- total + abs(abs(sample[i]-sample[j])-pi)-
(pi/2)
	total <- total - (2.895*(abs(sin(sample[i]-sample[j]))-(2/pi)))}}
		T <- total/n
		return(T)}
HermansRasson2PDeg <- function(sample){
		univals <- 9999
		n <- length(sample)
		sample<-circular(sample)
		sample <- rad(sample)
		testset<- rep(0,univals)
		for (f in 1:univals){
			data1 <- matrix(rcircularuniform(n,
control.circular=list(units="radians")))
			testset[f] <- HermansRasson2T(data1)}
		Tsample <- HermansRasson2T(sample)
		counter <- 1
		for(j in 1:univals){if(testset[j]>=Tsample){counter <- counter+1}}
		p <- counter/(univals+1)
		return(p)}
```

#Setting function for Pycke test

```
PyckeT <- function(sample){
		n <- length(sample)
		total <- 0
		for (i in 2:n){
			for (j in 1:(i-1)){
				numerator <- cos(sample[i]-sample[j])-sqrt(0.5)
				denominator <- 1.5-(2*sqrt(0.5)*cos(sample[i]-
sample[j]))
				total <- total + (numerator/denominator)}}
		T <- (2*total)/(n-1)
		return(T)}

PyckePDeg <- function(sample){
```

```
        univals <- 9999
        n <- length(sample)
        sample<-circular(sample)
        sample <- rad(sample)
        testset<- rep(0,univals)
        for (f in 1:univals){
                data1 <- matrix(rcircularuniform(n,
control.circular=list(units="radians")))
                testset[f] <- PyckeT(data1)}
        Tsample <- PyckeT(sample)
        counter <- 1
        for(j in 1:univals){if(testset[j]>=Tsample){counter <- counter+1}}
        p <- counter/(univals+1)
        return(p)}
```

#Preparing a test sample (von Mises) with a given sample size "n" and kappa

```
n <- 12
kappa <- 0.8
sampletest <- rvonmises(n, circular(0), kappa, control.circular=list(units="degrees"))
```

#Applying the rayleigh test

```
rayleigh.test(sampletest)
```

#applying the HR infinity test

```
HermansRassonPDeg (sampletest)
```

#applying the HR test

```
HermansRasson2PDeg (sampletest)
```

#applying the Pycke test

```
PyckePDeg (sampletest)
```

3. R code for sample generation for the different scenarios

# packages needed

library(circular)

library(NPCirc)

############ Random distribution

#Type 1 error - random sample - nv is the placeholder for the required sample size

RandomDistribution <- rcircmix(nv,model = 1)

############ Unimodal distribution

#Unimodal Skew Normal Distribution - nv is the placeholder for the required sample size, m1 for mean direction, k for Rho (dispersion parameter)

UnimdalSkewNormalDistribution <-rcircmix(nv,model = NULL, dist = c("wsn"),param=list(p=c(1), mu=c(m1), con=c(k),sk=c(30)))

#Unimodal Von Mises Distribution- nv is the placeholder for the required sample size, m1 for mean direction, k for kappa

UnimodaVonMisesDist <- rcircmix(nv,model = NULL, dist = c("vm"), param=list(p=c(1), mu=c(m1), con=c(k)))

############ Multimodal distribution with equal densities for each mode

#Bimodal Skew Normal Distribution - nv is the placeholder for the required sample sizes, m1& m2 for mean directions , k for Rho (dispersion parameter)

# mean directions for symmetrical distributions

m1=rad(360); m2=rad(180)

# mean directions for asymmetrical distributions

m1=rad(360); m2=rad(90)

BimodalSkewNormalDistribution <- rcircmix(nv,model = NULL, dist = c("wsn","wsn"),param=list(p=c(0.5,0.5), mu=c(m1,m2), con=c(k,k),sk=c(30,30)))

#Bimodal Von Mises Distribution- nv is the placeholder for the required sample sizes, m1&m2 for mean directions, k for kappas

# mean directions for symmetrical distributions

m1=rad(360); m2=rad(180)

# mean directions for asymmetrical distributions

m1=rad(360); m2=rad(90)

BimodalVonMisesDist <- rcircmix(nv,model = NULL, dist = c("vm","vm"),param=list(p=c(0.5,0.5), mu=c(m1,m2), con=c(k,k)))

#Trimodal Skew Normal Distribution - nv is the placeholder for the required sample sizes, m1 & m2 & m3 for mean directions , k for Rho (dispersion parameter)

# mean directions

m1=rad(360); m2=rad(120); m3=rad(240)

TrimodalSkewNormalDistribution<- rcircmix(nv,model = NULL, dist = c("wsn","wsn","wsn"),param=list(p=c(0.33333,0.33333,0.33333), mu=c(m1,m2,m3), con=c(k,k,k),sk=c(30,30,30)))

#Trimodal Von Mises Distribution- nv is the placeholder for the required sample sizes, m1 & m2 & m3 for mean directions, k for kappas

# mean directions

m1=rad(360); m2=rad(120); m3=rad(240)

TrimodalVonMisesDist <- rcircmix(nv,model = NULL, dist = c("vm","vm","vm"),param=list(p=c(0.33333,0.33333,0.33333), mu=c(m1,m2,m3), con=c(k,k,k)))

#Quadramodal Skew Normal Distribution - nv is the placeholder for the required sample sizes, m1 & m2 & m3 & m4 for mean directions , k for Rho (dispersion parameter)

#mean directions

m1=rad(360);m2=rad(90);m3=rad(180);m4=rad(270)

QuadramodalSkewNormalDistribution<-rcircmix(nv,model = NULL, dist = c("wsn","wsn","wsn","wsn"),
param=list(p=c(0.25,0.25,0.25,0.25), mu=c(m1,m2,m3,m4), con=c(k,k,k,k), sk=c(30,30,30,30)))

#Quadramodal Von Mises Distribution- nv is the placeholder for the required sample sizes, m1 & m2 & m3 & m4 for mean directions, k for kappas

# mean directions

```
m1=rad(360);m2=rad(90);m3=rad(180);m4=rad(270)
QuadramodalVonMisesDist <- rcircmix(nv,model = NULL, dist = c("vm","vm","vm","vm"),param=list(p=c(0.25,0.25,0.25,0.25),mu=c(m1,m2,m3,m4),con=c(k,k,k,k)))
```

####################

#For distributions with unequal densities between different modes we changed the p=c() argument in the param list.

#For bimodal: p=c(0.25,0.75)

#For trimodal: p= c(0.6,0.2,0.2)

#For quadramodal: p=c(0.4,0.2,0.2,0.2)

4. **Example for applying the tests in our simulations in R**

Type 1 error script for R

```
#Loading libraries used in script
library(compiler)
library(circular)
library(NPCirc)

#setting options for script performance
options("scipen" = 10)
enableJIT(1)

#Set function for HR infinity

HermansRassonTunc <- function(sample){
n <- length(sample)
total <- 0
for (i in 1:n){
for (j in 1:n){total <- total + abs(sin(sample[i]-sample[j]))}}
T <- abs((n/pi)-(total/(2*n)))
return(T)}

#Set function for HR

HermansRasson2Tunc <- function(sample){
n <- length(sample)
total <- 0
for (i in 1:n){
for (j in 1:n){ total <- total + abs(abs(sample[i]-sample[j])-pi)-(pi/2)
total <- total - (2.895*(abs(sin(sample[i]-sample[j]))-(2/pi)))}}
T <- total/n
return(T)}

HermansRassonT <- cmpfun(HermansRassonTunc)
HermansRasson2T <- cmpfun(HermansRasson2Tunc)
```

#Set function for Pycke test

```
PyckeTest <- function(sample){
  n <- length(sample)
  total <- 0
  for (i in 2:n){
    for (j in 1:(i-1)){
      numerator <- cos(sample[i]-sample[j])-sqrt(0.5)
      denominator <- 1.5-(2*sqrt(0.5)*cos(sample[i]-sample[j]))
      total <- total + (numerator/denominator)}}
  T <- (2*total)/(n-1)
  return(T)}

PyckeT <- cmpfun(PyckeTest)
```

#Set number of iterations, Sample size mean and kappa (or a sequence of any of that)

```
rans = 10000
nvseq = seq(10,120,10)
l = length(nvseq)
```

#Set Null samples to calculate p-values for HR infinity and HR

```
rand= 9999
Random <- sapply(1:rand, function(x)rcircularuniform(nv, control.circular=list(units="radians")))

RandomT <-as.vector(apply(Random, 2,HermansRassonT))
Random2T <-as.vector(apply(Random, 2,HermansRasson2T))
RandomPycke <-as.vector(apply(Random, 2,PyckeT))
```

#Prepare data frame for simulation results

```
Power.sample <- data.frame(matrix(nrow = l, ncol = 4))
names(Power.sample) <- c('ray',"HRinf", "HR",'Pycke’)
```

# For-loop to run the tests on 10000 random distributions

```
for(e in 1:l){
```

```
nv = nvseq[e]
###### set the distribution
  Distribution <- circular(sapply(1:rans, function(x)rcircmix(nv,model = 1)),units = 'radians')

# apply the tests
  Rayleigh <- (apply(Distribution,2,rayleigh.test))
  Ray <- array(as.matrix(unlist(Rayleigh)), dim=c(4, rans))
  RayP<- Ray[2,]

  HRT <- as.vector(apply(Distribution, 2, HermansRassonT))
HRTp <- sapply(HRT, function(x) sum(1*(RandomT >= x))/rand)
HRT2 <- as.vector(apply(Distribution, 2, HermansRasson2T))
HRT2p <- sapply(HRT2, function(x) sum(1*(Random2T >= x))/rand)

  Pyckres <- as.vector(apply(Distribution,2, PyckeT))
  Pyckep <- sapply(Pyckres, function(x) sum(1*(RandomPycke >= x))/rand)

  #Calculate p-value and store in Power.sample file
  Power.sample$ray[e]<- sum(1*(RayP< 0.05))/rans
  Power.sample$HRinf[e]<- sum(1*(HRTp < 0.05))/rans
  Power.sample$HR[e]<- sum(1*(HRT2p < 0.05))/rans
  Power.sample$Pycke[e]<- sum(1*(Pyckep < 0.05))/rans
} #e

#Power.sample now contains the results of the simulation of random data and can be viewed and exported
```
